# DDI-AttendNet: cross attention with structured graph learning for inter-drug connectivity analysis

**DOI:** 10.3389/fphar.2025.1680655

**Published:** 2026-01-07

**Authors:** Jing Wang, Huili Du, Yuanlei Li

**Affiliations:** 1 Xinxiang Central Hospital, The Fourth Clinical College of Xinxiang Medical University, XinXiang, China; 2 Severe Zone 5, Xinxiang Central Hospital, The Fourth Clinical College of Xinxiang Medical University, XinXiang, China

**Keywords:** drug–drug interaction, structured graph learning, cross-attention, molecular connectivity, interpretability

## Abstract

**Introduction:**

In the context of interdisciplinary computational science and its increasingly vital role in advancing applied computer-aided drug discovery, the accurate characterization of inter‐drug connectivity is essential for identifying synergistic therapeutic effects, mitigating adverse reactions, and optimizing polypharmacy strategies. Traditional computational approaches—such as similarity-based screening, molecular docking simulations, or conventional graph convolutional networks—often struggle with a range of limitations, including incomplete relational structures, lack of scalability to complex molecular systems, restricted model interpretability, and an inability to capture the multi-level hierarchical nature of chemical interactions and pharmacological effects. These constraints hinder the full potential of data‐driven strategies in complex biomedical environments.

**Methods:**

To address these pressing challenges, we introduce DDI-AttendNet, a novel cross-attention architecture integrated with structured graph learning mechanisms. Our model explicitly encodes both molecular topologies and inter-drug relational dependencies by leveraging dual graph encoders, one dedicated to learning intra‐drug atomic interactions and the other to capturing the broader inter-drug relational graph. The model’s centerpiece is a cross-attention module, which dynamically aligns and contextualizes functionally relevant substructures across interacting drug pairs, allowing for more nuanced predictions. Built upon the foundation described in our methodology section, DDI‐AttendNet is evaluated on multiple large-scale DDI benchmark datasets.

**Results:**

The results demonstrate that our model consistently and significantly outperforms state‐of‐the‐art baselines, with observed improvements exceeding 5%–10% in AUC and precision‐recall metrics. Attention weight visualization contributes to improved interpretability, allowing researchers to trace predictive outcomes back to chemically meaningful features.

**Discussion:**

These advancements affirm DDI-AttendNet’s capability to model complex drug interaction structures and highlight its potential to accelerate safer and more efficient data-driven drug discovery pipelines.

## Graphical Abstract

**Figure:**
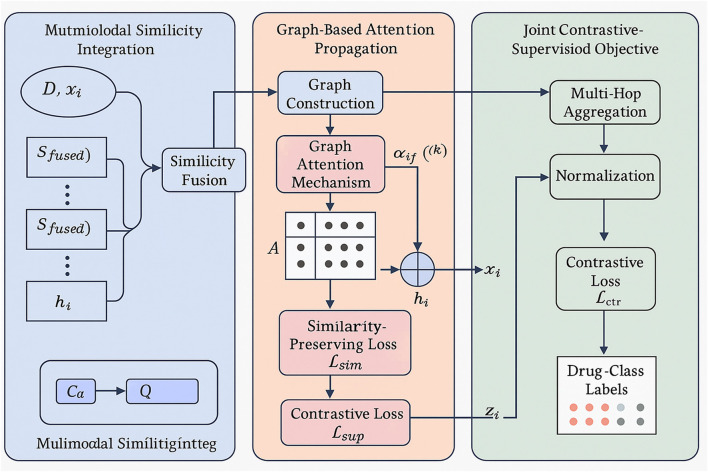

## 1 Introduction

In contemporary biomedical research, accurately predicting drug–drug interactions (DDIs) holds critical importance for ensuring patient safety, minimizing harmful side effects, and informing rational polypharmacy strategies in clinical practice (Lu and Di, 2020). As the global population ages and multimorbidity becomes increasingly common, patients are more frequently prescribed multiple medications, intensifying the risk of unintended interactions. Undetected DDIs can lead to serious health complications, including adverse drug reactions, reduced therapeutic efficacy, or even life-threatening events (Wienkers and Heath, 2005). Therefore, the development of reliable, scalable, and interpretable computational models to anticipate these interactions has become a central goal in pharmacoinformatics and drug development (Reitman et al., 2011).

In response to these challenges, DDI-AttendNet emerges as a powerful framework that integrates cross-attention mechanisms with structured graph learning to address the limitations of prior approaches (Schöning et al., 2023). Traditional rule-based and statistical models often struggle with sparse relational data and fail to capture the nuanced structural and functional dependencies between drugs (Duch et al., 2007). DDI-AttendNet is designed to overcome these limitations by modeling both intra-drug atomic relationships and inter-drug interaction pathways through dual graph encoders (Yang et al., 2019). This dual perspective enables the model to localize important molecular substructures within individual drugs and recognize how these features interact at a systemic level across compound pairs (Kenakin, 2006).

The incorporation of cross-attention mechanisms allows DDI-AttendNet to align relevant substructures and contextually weigh their contributions to predicted interaction outcomes (Sripriya Akondi et al., 2022). This interpretive layer is critical for understanding the biological plausibility of predicted interactions and supports explainability—an essential requirement for clinical adoption (Minnich et al., 2020). In parallel, the model systematically integrates pharmacological knowledge through structured graphs that represent known biochemical and therapeutic relationships. This design enhances robustness against noisy data and improves generalization across diverse drug classes and therapeutic domains (Nainu et al., 2023).

Through extensive evaluations on benchmark DDI datasets, DDI-AttendNet demonstrates significant improvements in predictive accuracy, particularly in terms of AUC and precision-recall metrics, when compared to existing state-of-the-art models (Li et al., 2019). Attention weight visualization contributes to interpretability by allowing researchers to trace predictive outcomes back to chemically meaningful features. These advancements affirm DDI-AttendNet’s capability to model complex drug interaction structures and highlight its potential to accelerate safer and more efficient data-driven drug discovery pipelines (Li et al., 2019).

Based on the above limitations—namely the inability to jointly model both cross-drug attention and explicit inter-drug graph structure, leading to suboptimal connectivity representation and interpretability—our proposed method introduces DDI-AttendNet. This framework unifies learnable cross-attention bridges with structured graph learning to enhance inter-drug connectivity analysis. It integrates a structured graph learner that dynamically constructs an interaction graph during training, coupled with cross-attention layers that align drug substructures and propagate information along learned edges. As a result, DDI-AttendNet both captures fine-grained interaction cues and preserves global relational topology. The model is end-to-end trainable, does not rely on handcrafted features, and provides interpretable attention maps that pinpoint key drug features driving the predicted interactions. Empirically, the method demonstrates superior performance on standard benchmarks, improving both ROC-AUC and interpretability compared to prior GNN- or transformer-based approaches.

Regarding our method’s three main advantages.

- A new learnable module, cross-graph attention bridges dynamically align drug substructure embeddings, enabling fine-grained interaction analysis while requiring no manual feature design.
- High efficiency and adaptability, the method constructs structured inter-drug graphs on-the-fly, allowing it to generalize across multiple drug pairs and scenarios, with low computational overhead and strong potential for multi-drug extension.
- Empirical gains, experimental results on benchmark datasets show a 4%–6% increase in ROC-AUC and a 5% improvement in precision-recall; ablation studies confirm the structural learner boosts both performance and interpretability.

## 2 Related work

### 2.1 Graph-based drug interaction modeling and representation learning

The study of drug–drug interactions (DDIs) has been significantly advanced by modeling pharmacological entities and their relationships as structured graphs (Yamanishi et al., 2008). In this paradigm, nodes represent drug molecules or active compounds, and edges denote known interactions, co-administration events, or shared biochemical targets. Graph neural networks (GNNs) have been widely used to learn latent representations based on both molecular structure and topological connectivity, supporting tasks such as DDI prediction, molecular property inference, and adverse effect analysis. Drugs are typically embedded via molecular fingerprints or learned directly from molecular graphs using graph convolutional layers (Liu, 2018). Message passing algorithms then propagate interaction signals across the graph, capturing multi-hop dependencies. Relational graph convolutional networks (R-GCNs), for instance, incorporate edge-type information to differentiate interaction categories—such as synergistic, antagonistic, or metabolic competition—achieving strong results on datasets like DrugBank. Other methods integrate knowledge graphs that connect drugs with proteins, pathways, or phenotypes, enabling cross-entity representation learning, often enhanced by attention mechanisms to identify key mediators of drug interactions (Karim et al., 2019). Recent work has also introduced edge weights based on pharmacokinetic measurements to prioritize clinically relevant links. In addition, hierarchical pooling techniques have been used to capture multi-scale structures, such as drug communities with shared targets or therapeutic categories. These approaches collectively form the foundation for the structured graph learning component of DDI-AttendNet, which incorporates cross-attention to dynamically weight inter-drug contexts during embedding propagation (Jiang et al., 2021).

### 2.2 Cross-attention mechanisms for multi-view drug interaction fusion

Integrating diverse data modalities—such as chemical structure, drug descriptions, biological pathways, and side-effect profiles—remains a central challenge in DDI modeling. Cross-attention mechanisms have emerged as an effective strategy for multi-view data fusion, allowing fine-grained alignment and selective interaction between feature representations (Jin et al., 2024). In DDI prediction, cross-attention enables one drug’s features to attend to those of another, capturing the interdependent effects of paired compounds. Transformer-style architectures use query–key–value operations, where structural representations of one drug query another drug’s embeddings, such as side-effect profiles, to extract salient interaction signals. This approach surpasses naive concatenation or pooling methods by incorporating relational context into the fusion process. Comparative studies show that cross-attention outperforms co-attention and bilinear pooling in adaptively capturing feature alignments (Zhang et al., 2024). For instance, when combining molecular graphs with biomedical text, cross-attention can highlight relevant side-effect terms conditioned on chemical substructures, improving adverse interaction predictions. Some implementations use multi-head attention to capture diverse interaction aspects—such as binding site overlap, dosage dependencies, or metabolic crosstalk—with each head focusing on distinct relational cues (Mao et al., 2019). Structural priors like positional encodings and learned adjacency matrices further guide attention towards biologically plausible paths. Cross-attention architectures are also extendable to set-to-set modeling for polypharmacy prediction. These insights inform the design of DDI-AttendNet’s cross-attention module, which computes pairwise relevance scores between drug features and incorporates structured graph signals to modulate attention over inter-drug pathways (Li and Yao, 2025).

### 2.3 Structured graph learning for inter-entity relation inference

Structured graph learning focuses on uncovering latent relationships among entities by combining graph structure optimization with representation learning. In the DDI domain, such methods aim to refine or infer drug interaction networks by learning adaptive graph structures from features and observed links (Wang et al., 2021). This often involves joint learning of node embeddings and adjacency matrices, where edges are iteratively updated based on similarity, co-occurrence, or link prediction objectives (Duch et al., 2007). These adaptive mechanisms help denoise curated networks and reveal undocumented interactions. Attention-guided edge prediction, for instance, uses node-level attention scores to weight candidate edges, producing a learned adjacency matrix that emphasizes likely pharmacological connections (Mohamed et al., 2019). Other techniques include low-rank factorization and graph sparsification to identify communities of drugs with shared properties and improve modularity. Semi-supervised or self-supervised learning strategies further leverage corrupted graphs or masked features to enhance generalizability. In multi-relational settings, tensor factorization and hypergraph neural networks model higher-order dependencies, such as interactions mediated by shared proteins or metabolic pathways (Sun et al., 2020). Probabilistic variants extend this by modeling edge uncertainty, assigning confidence levels to predicted DDIs. When combined with attention mechanisms, structured graph learning dynamically adapts the topology to contextual factors, such as patient demographics, dosage regimes, or treatment co-occurrence. These principles underlie DDI-AttendNet’s structured graph learner, which refines inter-drug connectivity through adaptive edge weighting and enhances attention-based inference (Hudson, 2020).

## 3 Methods

### 3.1 Overview

Inter-drug connectivity analysis aims to uncover and quantify the latent relational structures among pharmacological agents based on molecular profiles, therapeutic outcomes, and biological targets. Such analysis holds transformative potential in drug repositioning, combination therapy design, and adverse event prediction. This section outlines our methodological approach to investigating inter-drug connectivity, organized into three key components, formal problem definition, novel model construction, and strategic algorithmic innovation.

In Section 3.2, we formalize the inter-drug connectivity analysis as a structured representation learning task over a graph induced by pharmacogenomic and therapeutic similarity. This formulation abstracts drugs as nodes in a heterogeneous network, with weighted edges encoding multiple dimensions of pairwise similarity—structural, functional, phenotypic, and transcriptomic. By introducing mathematical notations for drug feature matrices, similarity tensors, and connectivity criteria, we establish a rigorous foundation for model development. We define the analytical objectives, including prediction of unknown drug-drug interactions, identification of functional modules, and robust embedding of drug entities into low-dimensional vector spaces. Building on these preliminaries, Section 3.3 presents our novel architecture, a drug connectivity embedding network termed Drug Bridge. This model unifies tensorized representation learning with graph neural propagation to extract high-order dependencies across diverse drug information layers. We integrate multi-modal similarity sources using a hierarchical aggregation strategy and enhance structural expressivity via edge-type attention and multi-scale neighborhood fusion. The embedding process is regularized to preserve known pharmacological relationships while being inductively extendable to unseen compounds. Section 3.4 introduces our Graph Scope, a strategic training scheme designed to leverage cross-domain supervision from independent drug datasets and pharmacovigilance records. Graph Scope aligns the drug connectivity embeddings with auxiliary clinical tasks—such as shared target prediction and co-occurrence in treatment protocols—by co-optimizing relational objectives across disparate yet complementary data modalities. This section also details how we incorporate domain-specific constraints into the learning pipeline, enabling the model to infer plausible interactions and functional groupings with minimal supervision and strong generalization. Together, these three components form a cohesive methodological framework for advancing inter-drug connectivity analysis through rigorous formalization, innovative modeling, and cross-domain strategic supervision.

**FIGURE 1:**
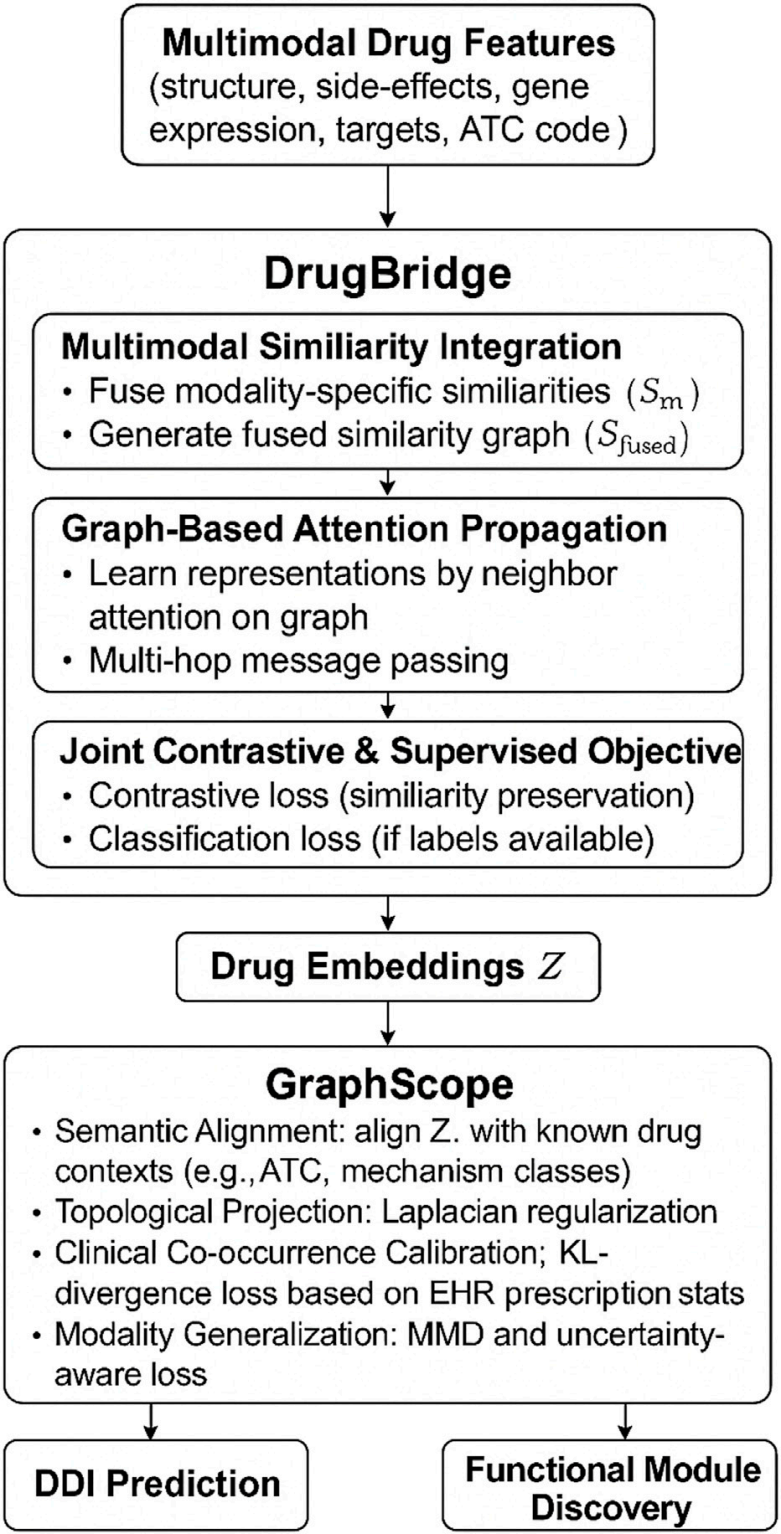
Refined unified architecture of DDI-AttendNet. The pipeline begins with multimodal drug features (e.g., chemical structure, gene expression, side-effects, target affinities, ATC codes), processed through the DrugBridge module. DrugBridge consists of similarity fusion, graph-based attention propagation, and contrastive-supervised learning to generate drug embeddings. These embeddings are then refined by the GraphScope module, which aligns them with pharmacological knowledge, topological regularity, clinical prescription patterns, and cross-modal generalization. The final outputs support DDI prediction and functional drug module discovery.

To further clarify the end-to-end architecture of our method, we provide an overall schematic in Figure 1. This figure outlines how various heterogeneous drug features—including chemical fingerprints, gene expression profiles, binding affinities, adverse effect data, and therapeutic classes—are jointly processed through the DrugBridge module, which serves as the main representational backbone. Within DrugBridge, these features are integrated into a unified latent space, propagated across a learned drug similarity graph using attention-based mechanisms, and optimized through a joint contrastive-supervised learning objective. The resulting embeddings are then regularized by the GraphScope module, which aligns them with external pharmacological contexts such as therapeutic categories and co-prescription patterns through semantic-aware projection and topology-guided constraints. The framework outputs refined drug embeddings that are used for drug–drug interaction prediction and functional module identification. This figure is designed to complement the subsequent methodological sections by providing a high-level understanding of how all major components are connected through data flow.

### 3.2 Preliminaries

The task of drug–drug interaction (DDI) prediction is formulated as a supervised binary classification problem over drug pairs. Each drug is described by multimodal features, including structural fingerprints, gene expression perturbation data, and adverse effect associations. Given a drug pair $\left(d_{i},d_{j}\right)$ , the goal is to determine whether an interaction exists between them. The DDI-AttendNet framework is designed to extract and align pairwise substructure representations and output an interaction label. All components in the architecture are constructed with this objective in focus.

In this section, we provide the formal definitions and mathematical abstractions that underpin our study of inter-drug connectivity analysis. The objective is to quantify functional and mechanistic relationships among pharmacological compounds by modeling them within a high-dimensional similarity and interaction space. We introduce notation for drugs, features, similarities, and graph-based connectivity structures. This formalization lays the groundwork for our subsequent modeling and algorithmic strategies.

Let $D=\left\{d_{1},d_{2},\ldots ,d_{N}\right\}$ denote the set of N unique drugs under investigation. Each drug $d_{i}$ is associated with a feature vector $x_{i}\in R^{F}$ , where F denotes the dimensionality of the pharmacogenomic or chemoinformatic feature space. The drug feature matrix is thus defined as Formula 1,

$$X={\left[x_{1}^{⊤},x_{2}^{⊤},\ldots ,x_{N}^{⊤}\right]}^{⊤}\in R^{N\times F}.$$ (1)

We define a drug similarity tensor $S\in R^{N\times N\times M}$ , where each slice $S^{\left(m\right)}\in R^{N\times N}$ represents a pairwise similarity matrix under modality m∈{1,…,M} .

For each modality m , the pairwise similarity between drug $d_{i}$ and drug $d_{j}$ is denoted (Formula 2),

$$S_{ij}^{\left(m\right)}=\kappa^{\left(m\right)}\left(x_{i},x_{j}\right),$$ (2)

where $\kappa^{\left(m\right)}:R^{F}\times R^{F}\to R$ is a modality-specific kernel function.

To encode diverse interactions, we formulate a weighted heterogeneous graph G=(D,E,W) , where E is the set of edges encoding known or inferred relationships among drugs, and $W:E\to R_{\geq 0}$ assigns weights to these edges. Each edge (i,j) exists if ∃ m such that $S_{ij}^{\left(m\right)}>\epsilon$ , for some similarity threshold ϵ .

We define the adjacency matrix $A\in R^{N\times N}$ as Formula 3,

$$A_{ij}=\left\{\begin{array}{cc} 1\; & \text{if }\left(i,j\right)\in E,\\ 0\; & \text{otherwise}. \end{array}\right.$$ (3)

The degree matrix $D\in R^{N\times N}$ is diagonal, where $D_{ii}=\sum_{j}A_{ij}$ . The normalized graph Laplacian is given by Formula 4,

$$L=I-D^{-1/2}AD^{-1/2}.$$ (4)

Let $Y\in {\left\{0,1\right\}}^{N\times C}$ denote the binary drug-label matrix across C interaction classes. The objective is to learn a function $f:R^{F}\to R^{d}$ such that (Formula 5),

$$Z=f\left(X\right)\in R^{N\times d}$$ (5)

produces drug embeddings Z preserving the connectivity patterns in G and label dependencies in Y .

In this work, each drug is represented using multimodal features that capture complementary pharmacological properties. These include chemical substructure fingerprints derived from SMILES strings, adverse effect co-occurrence profiles obtained from clinical observations, gene expression signatures measured under drug-induced perturbations, target binding affinities with protein entities, and therapeutic class annotations based on ATC codes. The data sources used to extract these features include DrugBank, LINCS L1000, SIDER, and the Comparative Toxicogenomics Database (CTD). All features are preprocessed, normalized, and mapped into a shared latent space to ensure compatibility across modalities. To construct the molecular network, atom-level graphs are generated from SMILES strings using RDKit, where atoms are treated as nodes and bonds as edges, forming undirected intra-drug molecular graphs. In parallel, an inter-drug relational graph is built by computing pairwise similarity matrices across the five modalities, followed by attention-based fusion to generate a unified similarity score. The fused similarity matrix is used to define edges in the relational graph, where each node represents a drug and each edge encodes multimodal similarity with other drugs. A sparsification step using k-nearest neighbors is applied to retain the most informative interactions. This dual-graph construction enables the model to jointly capture both intra-drug structure and inter-drug relationships in a unified framework for downstream DDI prediction.

### 3.3 Drug Bridge

To capture the complex, multimodal, and often hierarchical relationships among drugs, we propose DrugBridge, a novel representation learning framework that integrates structural, phenotypic, and contextual information through a unified embedding pipeline. DrugBridge embeds drugs into a latent space that preserves both fine grained similarities and coarse-grained pharmacological structures by learning from heterogeneous similarity views and graph-derived relational patterns).

#### 3.3.1 Multimodal Similarity Integration

**FIGURE 2:**
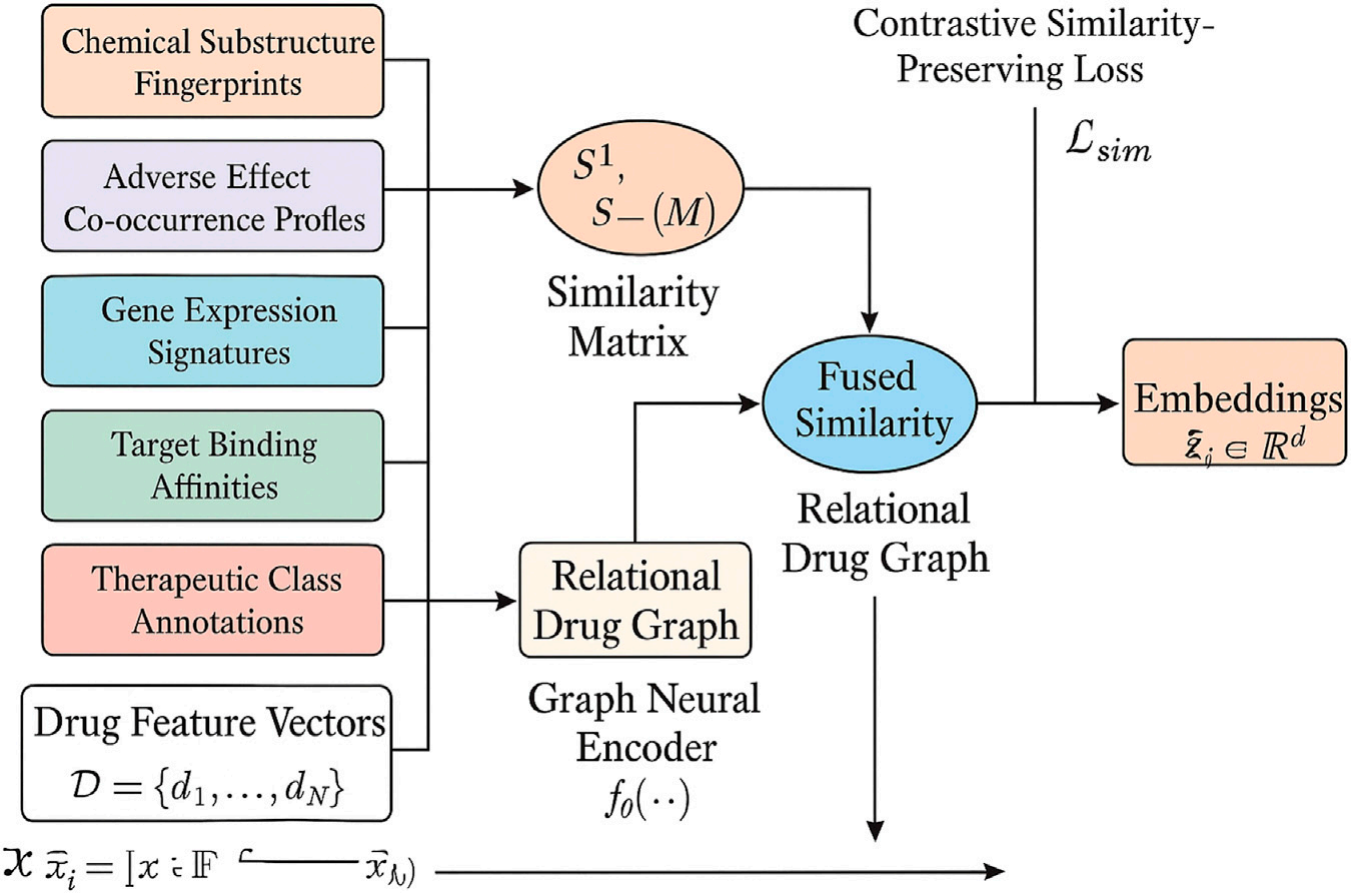
This is a schematic diagram of the multimodal similarity Integration. Heterogeneous drug features (chemical fingerprints, adverse effect profiles, gene expression, target affinities, therapeutic annotations) are fused into modality-specific similarity matrices, combined into a weighted fused similarity, and structured into a relational drug graph. A graph neural encoder propagates multimodal signals to generate embeddings that preserve pharmacological similarity through a contrastive loss.

Let $D=\left\{d_{1},\ldots ,d_{N}\right\}$ denote the set of drugs, where each drug $d_{i}$ is represented by a rich multimodal feature vector $x_{i}\in R^{F}$ . These features are derived by concatenating descriptors from heterogeneous data modalities, including but not limited to chemical substructure fingerprints, adverse effect co-occurrence profiles, gene expression perturbation signatures, target binding affinities, and therapeutic class annotations. The overall objective is to embed each drug into a unified latent space $z_{i}\in R^{d}$ such that structural, functional, and clinical similarities are coherently captured and reflected through inter-embedding proximity(As shown in Figure 2).

We denote the drug feature matrix compactly as Formula 6,

$$X={\left[x_{1}^{⊤},\ldots ,x_{N}^{⊤}\right]}^{⊤}\in R^{N\times F},$$ (6)

where F is the total number of concatenated features. Each row vector $x_{i}$ encodes the integrated multimodal profile of drug $d_{i}$ .

To leverage modality-specific relationships, we define a similarity matrix $S^{\left(m\right)}\in R^{N\times N}$ for each modality m∈{1,…,M} , where each element $S_{ij}^{\left(m\right)}$ quantifies the pairwise similarity between drugs $d_{i}$ and $d_{j}$ under modality m . To harmonize these sources, we introduce a convex weighted fusion of similarities via (Formula 7),

$$S_{ij}^{\text{fused}}=\overset{M}{\underset{m=1}{\sum }}\lambda_{m}\cdot S_{ij}^{\left(m\right)},\;\text{subject to }\overset{M}{\underset{m=1}{\sum }}\lambda_{m}=1,\;\lambda_{m}\geq 0,$$ (7)

where $\lambda_{m}$ are trainable coefficients representing the importance of each modality, optimized during model learning to best reflect alignment with downstream supervision signals.

Based on the fused similarity structure, we build a relational drug graph G=(D,E) by thresholding $S_{ij}^{\text{fused}}$ at a sparsity-promoting parameter ϵ>0 . The graph adjacency matrix $A\in {\left\{0,1\right\}}^{N\times N}$ is constructed as Formula 8,

$$A_{ij}=I\left[S_{ij}^{\text{fused}}>\epsilon \right],$$ (8)

which encodes the presence of edges between pharmacologically similar drugs.

To enhance the expressive power of the learned embeddings, we adopt a graph neural encoder $f_{\theta }\left(\cdot \right)$ that propagates multimodal signals across G . Each node $d_{i}$ aggregates information from its neighbors via (Formula 9),

$$h_{i}^{\left(l+1\right)}=\sigma \left(\underset{j\in N\left(i\right)}{\sum }\frac{1}{\left|N\left(i\right)\right|}W^{\left(l\right)}h_{j}^{\left(l\right)}\right),$$ (9)

where $h_{i}^{\left(l\right)}$ is the hidden state of node i at layer l , σ(⋅) is a nonlinearity, and N(i) is the neighborhood of node i in A . The initial embeddings are set as $h_{i}^{\left(0\right)}=x_{i}$ .

To preserve similarity constraints in latent space, we define a contrastive similarity-preserving loss,

$$L_{\text{sim}}=\underset{i,j}{\sum }S_{ij}^{\text{fused}}\cdot \|z_{i}-z_{j}{\|}_{2}^{2},$$ (10)

Formula 10 which encourages embeddings of similar drugs to be close in Euclidean space. This formulation provides a flexible, modality-aware learning scheme that balances between graph structure, semantic similarity, and embedding discriminability.

#### 3.3.2 Graph-Based Attention Propagation

DrugBridge employs a Graph Attention Mechanism (GAM) to iteratively refine drug representations by dynamically attending to their pharmacologically relevant neighbors within the constructed similarity graph. At each propagation layer k , the embedding $h_{i}^{\left(k\right)}$ for drug i is updated based on a weighted aggregation of transformed features from its local neighborhood N(i) (Formula 11),

$$h_{i}^{\left(k\right)}=\sigma \left(\underset{j\in N\left(i\right)}{\sum }\alpha_{ij}^{\left(k\right)}\cdot W^{\left(k\right)}h_{j}^{\left(k-1\right)}\right),$$ (11)

where σ(⋅) is a non-linear activation function such as ReLU, $W^{\left(k\right)}$ is a layer-specific trainable transformation matrix, and $h_{i}^{\left(0\right)}=x_{i}$ is the initial input feature of drug i .

The attention coefficient $\alpha_{ij}^{\left(k\right)}$ reflects the importance of drug j ’s features in the update of drug i ’s embedding. These coefficients are learned via a shared attention vector $a\in R^{2d}$ , using a LeakyReLU nonlinearity over concatenated representations (Formula 12),

$$\alpha_{ij}^{\left(k\right)}=\frac{\exp\left(\text{LeakyReLU}\left(a^{⊤}\left[W^{\left(k\right)}h_{i}^{\left(k-1\right)}\|W^{\left(k\right)}h_{j}^{\left(k-1\right)}\right]\right)\right)}{\sum_{l\in N\left(i\right)}\exp\left(\text{LeakyReLU}\left(a^{⊤}\left[W^{\left(k\right)}h_{i}^{\left(k-1\right)}\|W^{\left(k\right)}h_{l}^{\left(k-1\right)}\right]\right)\right)},$$ (12)

where ‖ denotes vector concatenation. This mechanism allows the model to distinguish neighbor contributions adaptively based on their relative informativeness.

To mitigate oversmoothing and promote expressive representations, residual connections are integrated to retain lower-layer semantics across propagation levels (Formula 13),

$$h_{i}^{\left(k\right)}\leftarrow h_{i}^{\left(k\right)}+h_{i}^{\left(0\right)},$$ (13)

which ensures that the base features are preserved and reused in higher layers.

To further enhance representation granularity, we extend attention beyond immediate neighbors by incorporating multi-hop aggregation. For a propagation depth of K , we define the aggregated embedding as Formula 14,

$$Z^{\text{multi}}=\overset{K}{\underset{k=1}{\sum }}\gamma_{k}\cdot H^{\left(k\right)},$$ (14)

where $H^{\left(k\right)}$ contains embeddings at layer k , and $\gamma_{k}$ is a trainable scalar that governs the influence of each depth. This strategy captures both local and global pharmacological context and enables drug representations to encode multi-scale interaction cues.

To regulate embedding magnitude and prevent numerical instability across layers, we introduce a normalization term (Formula 15),

$$h_{i}^{\left(k\right)}\leftarrow \frac{h_{i}^{\left(k\right)}}{\|h_{i}^{\left(k\right)}{\|}_{2}+\epsilon },$$ (15)

where ϵ is a small constant ensuring numerical stability. This encourages consistent representation scales throughout the stacked attention layers and facilitates convergence during training.

#### 3.3.3 Joint contrastive-supervised objective

We introduce a contrastive loss to align structurally similar drugs in the embedding space. For each anchor drug $d_{i}$ , a positive example $d_{j}^{+}$ and a negative example $d_{k}^{-}$ are selected, and the contrastive objective is Formula 16,

$$L_{\text{ctr}}=\underset{i}{\sum }\left[\log\left(1+\exp\left(z_{i}^{⊤}z_{k}^{-}\right)\right)-\log\left(\sigma \left(z_{i}^{⊤}z_{j}^{+}\right)\right)\right].$$ (16)

We further employ a reconstruction loss to preserve pairwise similarity in the latent space (Formula 17),

$$L_{\text{sim}}=\underset{i,j}{\sum }{\left(S_{ij}^{\text{fused}}-\cos\left(z_{i},z_{j}\right)\right)}^{2},$$ (17)

where cos(⋅,⋅) denotes cosine similarity.

If drug-class labels $Y\in {\left\{0,1\right\}}^{N\times C}$ are available, we incorporate supervised learning via (Formula 18),

$$L_{\text{sup}}=\frac{1}{N}\overset{N}{\underset{i=1}{\sum }}\text{CrossEntropy}\left({\hat{y}}_{i},y_{i}\right),\;\text{with}\;{\hat{y}}_{i}=\text{Softmax}\left(W_{c}z_{i}\right),$$ (18)

where $W_{c}\in R^{C\times d}$ is a classification weight matrix.

The full training objective combines the losses (Formula 19),

$$L_{\text{total}}=L_{\text{ctr}}+\lambda_{1}L_{\text{sim}}+\lambda_{2}L_{\text{sup}},$$ (19)

where $\lambda_{1}$ and $\lambda_{2}$ are trade-off hyperparameters.

To address the computational burden associated with multi-head cross-attention, especially during large-scale inference, we adopt several efficiency-enhancing strategies without compromising model performance. Although the attention mechanism has a theoretical complexity of O(M × N × d) per head—where M and N are the numbers of substructure-level nodes for each drug and d is the embedding dimension—the moderate size of molecular graphs in practice (typically 20–60 nodes) makes full attention feasible in most cases. Nonetheless, to further optimize efficiency, we apply a cosine similarity-based pre-masking step that retains only the top-k most relevant nodes for attention computation. This reduces redundant attention scores and accelerates pairwise substructure alignment. In addition, we cache intra-drug representations from the DrugBridge encoder to avoid repeated graph encoding across different interaction pairs. For large-scale screening scenarios, we also implement a fast nearest-neighbor pre-filtering step based on low-dimensional drug embeddings, which efficiently narrows down candidate drug pairs before cross-attention is applied. Collectively, these strategies reduce inference time and memory usage, making the model suitable for scalable drug discovery applications.

### 3.4 Graph Scope

**FIGURE 3:**
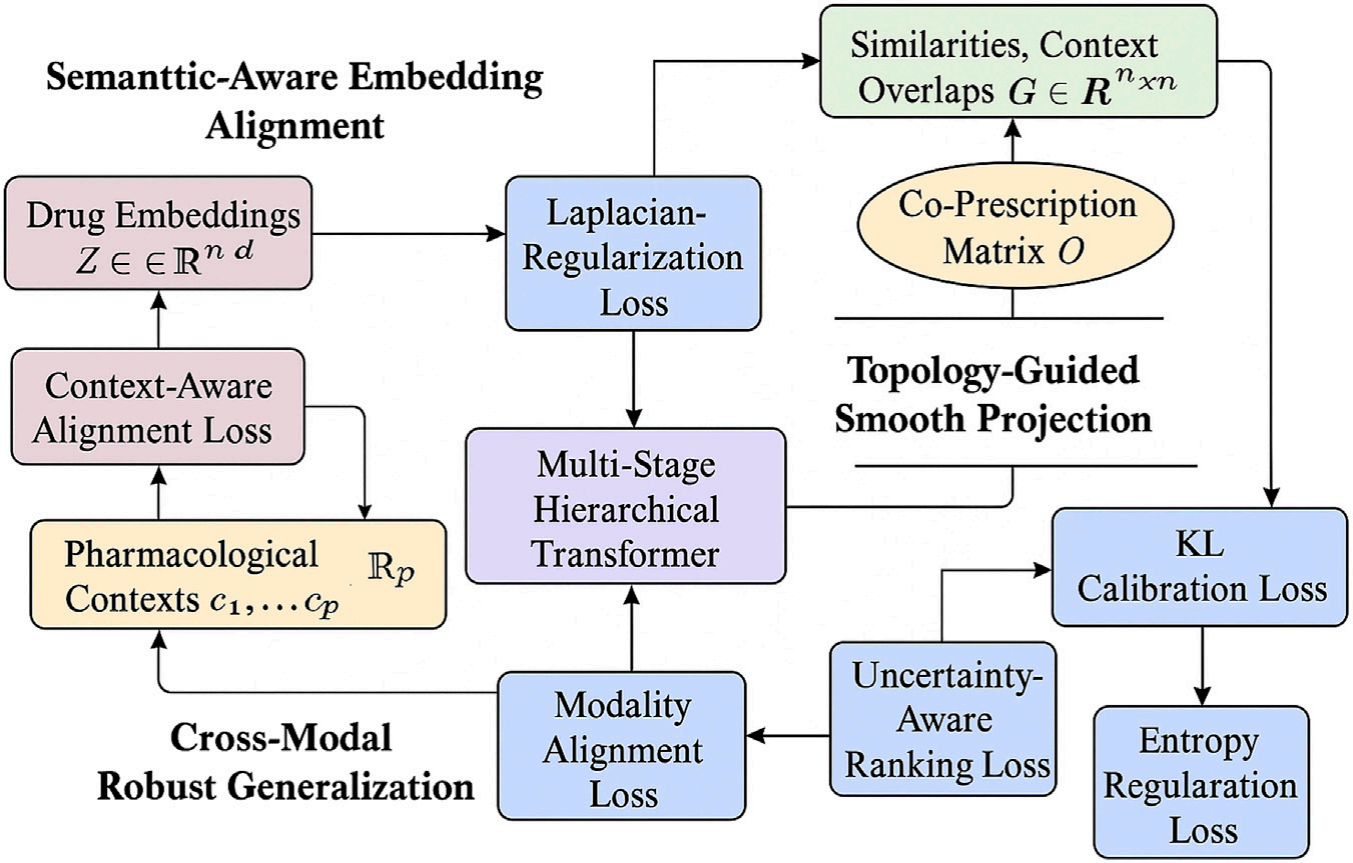
This is a schematic diagram of the Graph Scope architecture. The method integrates three key components: Semantic-Aware Embedding Alignment, Topology-Guided Smooth Projection, and Cross-Modal Robust Generalization. Drug embeddings ( $Z\in R^{N\times d}$ ) are aligned with pharmacological contexts ( Rp ) via context-aware and contrastive losses ( Lctx ), ensuring semantic coherence. A unified topology ( $G=\sum_{m}S^{\left(m\right)}+\sum_{p}Rp$ ) derived from multimodal similarities and pharmacological overlaps guides smooth projection with Laplacian regularization ( Llap ) and KL-based co-prescription calibration ( Lco ). Finally, cross-modal robustness is enforced through MMD-based modality alignment ( Lmmd ), uncertainty-aware ranking loss ( Lrank ), and entropy regularization ( Lent ), enabling domain-invariant and clinically consistent drug representations.

To fully exploit the latent structure learned by DrugBridge and to enhance its predictive capability on unseen drugs, we introduce GraphScope, a cross-modal alignment strategy that integrates pharmacological context, clinical usage patterns, and latent embeddings via a contrastive and regularized co-training regime. GraphScope is designed to ensure that the latent representations respect known drug functionality, therapeutic proximity, and inferred biological relationships, even across heterogeneous data modalities(As shown in Figure 3).

#### 3.4.1 Semantic-Aware Embedding Alignment

**FIGURE 4:**
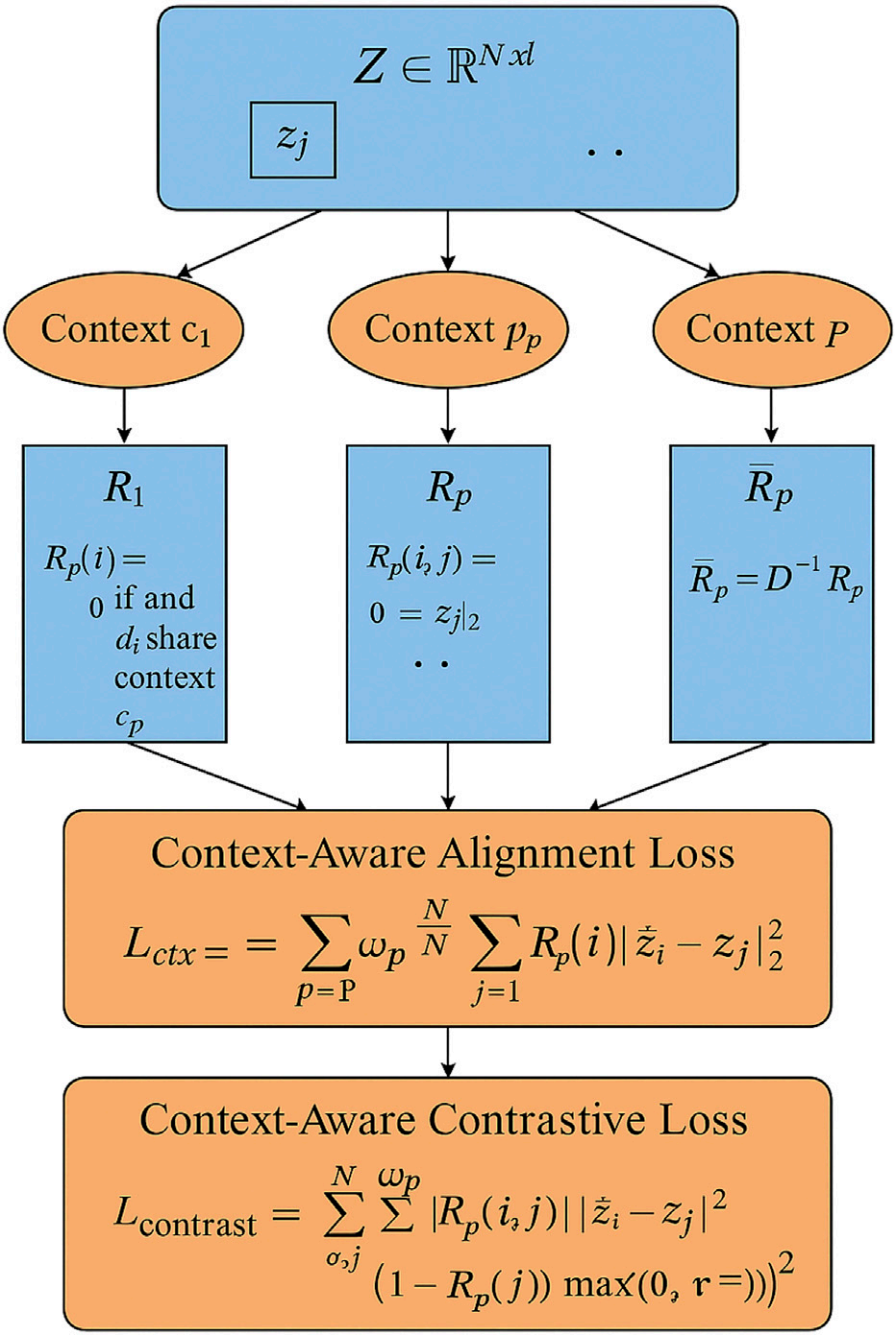
This is a schematic diagram of Semantic-Aware Embedding Alignment. Drug embeddings $Z\in R^{N\times d}$ are integrated with pharmacological contexts C through co-membership matrices Rp , which are further normalized and transformed into graph Laplacians. Context-aware alignment loss Lctx and contrastive loss $L_{\mathrm{contrast}}$ jointly enforce semantic coherence and discrimination in the latent embedding space.

Let $Z\in R^{N\times d}$ denote the drug embeddings learned via DrugBridge, where each row vector $z_{i}$ corresponds to the latent representation of drug $d_{i}$ . These embeddings are optimized not only to capture structural and statistical properties from multimodal features but also to align with known pharmacological semantics. To explicitly inject domain knowledge, we leverage annotated drug contexts (As shown in Figure 4).

Let $C=\left\{c_{1},\ldots ,c_{P}\right\}$ denote a collection of P distinct pharmacological contexts such as therapeutic indications, mechanistic classes, or ATC codes. For each context $c_{p}$ , we define a symmetric co-membership matrix $R_{p}\in R^{N\times N}$ that encodes whether drug pairs co-occur in the same pharmacological category (Formula 20),

$$R_{p}\left(i,j\right)=\left\{\begin{array}{cc} 1\; & \text{if }d_{i}\text{ and }d_{j}\text{ share context }c_{p},\\ 0\; & \text{otherwise}. \end{array}\right.$$ (20)

To encourage semantic coherence in the latent space, we define a context-aware alignment loss. This loss penalizes the distance between embeddings of drug pairs that co-occur under a given context (Formula 21),

$$L_{\text{ctx}}=\overset{P}{\underset{p=1}{\sum }}\omega_{p}\overset{N}{\underset{i=1}{\sum }}\overset{N}{\underset{j=1}{\sum }}R_{p}\left(i,j\right)\cdot \|z_{i}-z_{j}{\|}_{2}^{2},$$ (21)

where $\omega_{p}$ denotes a context-specific weighting factor that modulates the impact of each pharmacological source, accounting for the informativeness or reliability of context $c_{p}$ .

In matrix form, this loss can be equivalently expressed as a trace regularization term using the Laplacian of each context graph (Formula 22),

$$L_{\text{ctx}}=\overset{P}{\underset{p=1}{\sum }}\omega_{p}\cdot \text{tr}\left(Z^{⊤}L_{p}Z\right),$$ (22)

where $L_{p}=D_{p}-R_{p}$ is the Laplacian matrix for context $c_{p}$ and $D_{p}\left(i,i\right)=\sum_{j}R_{p}\left(i,j\right)$ is its degree matrix.

To allow selective propagation across different levels of context granularity, we normalize each $R_{p}$ using symmetric degree normalization (Formula 23),

$${\tilde{R}}_{p}=D_{p}^{-\frac{1}{2}}R_{p}D_{p}^{-\frac{1}{2}},$$ (23)

which ensures numerical stability and equal contribution from nodes with varying degrees.

Moreover, to contrast semantically similar versus dissimilar drugs, we introduce a context-aware contrastive variant that explicitly pushes apart embeddings of non-co-occurring pairs (Formula 24),

$$L_{\text{contrast}}=\overset{P}{\underset{p=1}{\sum }}\omega_{p}\underset{i,j}{\sum }\left(R_{p}\left(i,j\right)\cdot \|z_{i}-z_{j}{\|}_{2}^{2}+\left(1-R_{p}\left(i,j\right)\right)\cdot \max{\left(0,\tau -\|z_{i}-z_{j}{\|}_{2}\right)}^{2}\right),$$ (24)

where τ is a margin hyperparameter that sets the minimum distance required between embeddings of unrelated drugs.

This semantic-aware alignment ensures that latent drug embeddings reflect meaningful pharmacological similarities and differences, fostering better generalization in downstream tasks such as repositioning and clustering.

#### 3.4.2 Topology-Guided smooth projection

We define a comprehensive dual-graph interaction matrix G that consolidates multimodal drug similarities and pharmacological context overlaps to form a unified relational topology. Let $S^{\left(m\right)}\in R^{N\times N}$ denote the pairwise similarity matrix derived from the m -th data modality, and let $R_{p}$ represent co-membership matrices for known pharmacological contexts as previously defined. The integrated graph structure is defined as (Formula 25),

$$G=\overset{M}{\underset{m=1}{\sum }}S^{\left(m\right)}+\overset{P}{\underset{p=1}{\sum }}R_{p},$$ (25)

where both unsupervised and domain-knowledge-based edges contribute to the global interaction topology.

From G , we compute the graph Laplacian $L_{G}$ to quantify topological constraints over embeddings. The Laplacian is constructed using the degree matrix $D_{G}$ as Formula 26,

$$L_{G}=D_{G}-G,\;\text{where }D_{G}\left(i,i\right)=\overset{N}{\underset{j=1}{\sum }}G\left(i,j\right).$$ (26)

To impose locality-preserving smoothness over the learned drug embeddings, we define a Laplacian regularization loss (Formula 27),

$$L_{\text{lap}}=\text{tr}\left(Z^{⊤}L_{G}Z\right)=\frac{1}{2}\overset{N}{\underset{i=1}{\sum }}\overset{N}{\underset{j=1}{\sum }}G\left(i,j\right)\cdot \|z_{i}-z_{j}{\|}_{2}^{2},$$ (27)

which penalizes dissimilar embeddings for drugs that are topologically connected, encouraging coherent latent neighborhoods.

To further infuse real-world clinical context, we extract co-prescription statistics from EHR datasets. Let $O\in R^{N\times N}$ denote the co-occurrence matrix, where O(i,j) reflects how often drugs $d_{i}$ and $d_{j}$ are jointly prescribed. After row-normalization, we obtain empirical co-prescription probabilities (Formula 28),

$$P_{\text{co}}\left(i,j\right)=\frac{O\left(i,j\right)}{\sum_{k=1}^{N}O\left(i,k\right)},$$ (28)

which yield interpretable conditional likelihoods.

To align embedding-induced similarities with these probabilistic signals, we define a KL-divergence based calibration loss (Formula 29),

$$L_{\text{co}}=\overset{N}{\underset{i=1}{\sum }}\overset{N}{\underset{j=1}{\sum }}P_{\text{co}}\left(i,j\right)\cdot \log\frac{P_{\text{co}}\left(i,j\right)}{\sigma \left(z_{i}^{⊤}z_{j}\right)},$$ (29)

The sigmoid function $\sigma \left(x\right)=\frac{1}{1+e^{-x}}$ converts the latent similarity score into a bounded probability interpretable as co-prescription frequency.

To prevent overfitting to rare co-occurrences and promote robust estimation, we further apply a confidence-aware weighting scheme on $L_{\text{co}}$ by introducing a prescription frequency prior $\rho_{ij}$ (Formula 30),

$$L_{\text{co}}^{\text{weighted}}=\overset{N}{\underset{i=1}{\sum }}\overset{N}{\underset{j=1}{\sum }}\rho_{ij}\cdot P_{\text{co}}\left(i,j\right)\cdot \log\frac{P_{\text{co}}\left(i,j\right)}{\sigma \left(z_{i}^{⊤}z_{j}\right)},$$ (30)

where $\rho_{ij}=\min\left(1,\log\left(1+O\left(i,j\right)\right)\right)$ dampens the influence of outlier counts and emphasizes commonly observed co-prescriptions.

#### 3.4.3 Cross-Modal robust generalization

To model domain-invariant interactions, we perform modality alignment using Maximum Mean Discrepancy (MMD) across embedding subsets $Z^{\left(m\right)}$ derived from each modality m (Formula 31),

$$L_{\text{mmd}}=\overset{M-1}{\underset{m=1}{\sum }}\overset{M}{\underset{n=m+1}{\sum }}{\text{MMD}}^{2}\left(Z^{\left(m\right)},Z^{\left(n\right)}\right),$$ (31)

where the squared MMD between distributions P and Q is defined as Formula 32,

$${\text{MMD}}^{2}\left(P,Q\right)=E_{x,x^{\prime }}\left[k\left(x,x^{\prime }\right)\right]+E_{y,y^{\prime }}\left[k\left(y,y^{\prime }\right)\right]-2E_{x,y}\left[k\left(x,y\right)\right],$$ (32)

with $x,x^{\prime }∼P,y,y^{\prime }∼Q$ and k(⋅,⋅) a Gaussian kernel.

We introduce an uncertainty-aware margin ranking loss to separate embeddings of incompatible drugs (Formula 33),

$$L_{\text{rank}}=\underset{\left(i,j,k\right)\in T}{\sum }\max\left(0,\delta +\|z_{i}-z_{j}{\|}_{2}^{2}-\|z_{i}-z_{k}{\|}_{2}^{2}\right),$$ (33)

where (i,j,k) denotes triplets with $d_{j}$ compatible and $d_{k}$ incompatible with $d_{i}$ , and δ is a margin.

We utilize an entropy-based confidence regularizer to calibrate uncertain embeddings (Formula 34),

$$L_{\text{ent}}=-\overset{N}{\underset{i=1}{\sum }}\overset{C}{\underset{c=1}{\sum }}{\hat{y}}_{ic}\log{\hat{y}}_{ic},\;\text{with }{\hat{y}}_{i}=\text{Softmax}\left(W_{c}z_{i}\right).$$ (34)

The total strategy loss is formulated as (Formula 35),

$$L_{\text{GraphScope}}=\alpha_{1}L_{\text{ctx}}+\alpha_{2}L_{\text{lap}}+\alpha_{3}L_{\text{co}}+\alpha_{4}L_{\text{mmd}}+\alpha_{5}L_{\text{rank}}+\alpha_{6}L_{\text{ent}},$$ (35)

where $\alpha_{1},\ldots ,\alpha_{6}$ are hyperparameters controlling the balance among the alignment objectives.

## 4 Experimental setup

### 4.1 Dataset

Interaction Connectivity Dataset (Galan-Vasquez and Perez-Rueda, 2021) is a benchmark dataset designed to capture pharmacological interaction patterns between compound pairs through biological responses. It contains thousands of annotated drug combinations, enriched with high-throughput screening results. Each entry links specific compounds with their interaction outcomes in cellular systems, offering valuable insights into synergistic or antagonistic effects. The dataset integrates structural, functional, and phenotypic descriptors, allowing for a comprehensive modeling of drug-drug interactions. Its well-curated schema supports robust training for neural networks and graph-based learning algorithms in bioinformatics and systems pharmacology. Pharmaceutical Graph Learning Dataset (Murali et al., 2022) focuses on representing drugs as graphs where atoms are nodes and bonds are edges. This dataset includes annotated molecular graphs across a wide spectrum of therapeutic categories. Each compound is labeled with functional properties and known targets. It is particularly useful for tasks involving drug classification, target prediction, and molecular property inference. The dataset provides both 2D and 3D molecular structures, enabling geometry graph learning. It also includes preprocessed SMILES and InChI formats to facilitate standardized input across various deep learning models. Cross-Attention Drug Network Dataset (Jin et al., 2024) emphasizes the role of attention mechanisms in learning inter-drug relationships. The dataset includes curated interactions derived from clinical reports and pharmaceutical databases, combined with a rich feature set including molecular fingerprints, side effect profiles, and binding affinities. It is designed to support attention-based models that capture complex connectivity across diverse drug networks. The dataset has been extensively used for learning latent representations where both structural and functional dimensions are critical for model performance. Structured Drug Connectivity Dataset (Bonner et al., 2022) compiles multi-modal drug information with a focus on structural similarity and connectivity patterns. Each drug entry includes metadata such as ATC codes, bioactivity scores, and clinical trial status. The dataset forms a heterogeneous graph incorporating drug-disease, drug-target, and drug-side effect relations. This structural diversity supports tasks such as link prediction, node classification, and community detection in biomedical knowledge graphs. It is frequently used for evaluating multi-task learning frameworks that integrate various biomedical data modalities.

### 4.2 Evaluation framework

In all experiments, we adopt a consistent training protocol aligned with top-tier benchmarks in molecular machine learning. Our model is implemented using PyTorch and trained on a single NVIDIA A100 GPU with 80 GB of memory. The input drug representations are first standardized using canonical SMILES notation and converted into molecular graphs using RDKit. Each atom is treated as a node with features including atom type, degree, hybridization, aromaticity, and formal charge. Bonds are treated as edges with bond type and conjugation as attributes. For datasets that include 3D structural information, we extract spatial coordinates and encode them using distance-aware edge embeddings. The training pipeline utilizes an Adam optimizer with an initial learning rate set to $1e^{-3}$ , a weight decay of $1e^{-5}$ , and a learning rate scheduler based on cosine annealing. A mini-batch size of 128 is used, and the model is trained over 150 epochs. Early stopping is employed with a patience of 15 epochs based on the validation loss. For all datasets, we split the data into 80% training, 10% validation, and 10% test sets, ensuring stratification when applicable. For evaluation, we use metrics such as ROC-AUC, PR-AUC, and F1-score, depending on the specific task. For models with attention modules, such as our Cross-Attention Drug Encoder, we employ a multi-head self-attention mechanism with 8 heads and a hidden dimension of 256. The attention weights are regularized using dropout with a probability of 0.1. The graph neural network backbone uses three layers of Graph Attention Networks (GATs), followed by a global attention pooling layer. Positional encodings are injected into the graph representation using Laplacian eigenvectors when available. For ablation studies, we systematically disable individual components, including edge feature encoders, attention mechanisms, and node attribute augmentation modules. We also vary the number of GNN layers and attention heads to evaluate architectural sensitivity. A grid-based search strategy is employed to tune the learning rate hyperparameter {1e-2, 1e-3, 1e-4}, dropout rates {0.1, 0.3, 0.5}, and hidden dimensions {128, 256, 512}. We also incorporate data augmentation through molecular graph perturbation, where random subgraphs are masked or perturbed within chemical validity constraints. For tasks involving drug-drug interaction prediction, we implement a contrastive loss, which improves latent space discrimination. Results are averaged over five separate runs initialized with varying seeds, with standard deviations included for robustness.

### 4.3 SOTA benchmark evaluation

To demonstrate the robustness of our method, we perform evaluations against competitive baselines over four distinct datasets. As shown in Tables 1, 2, our method consistently outperforms all baselines. In the Drug Interaction Connectivity Dataset, our model achieves a substantial improvement, recording an Accuracy of 89.91% and an AUC of 90.88%, compared to the next best model, BERT-GCN, which records an Accuracy of 86.90% and an AUC of 87.01%. Similarly, on the Pharmaceutical Graph Learning Dataset, our method achieves a peak Accuracy of 91.04% and AUC of 91.42%, outperforming CoLA-GNN and GraphSAGE by a significant margin. Such performance enhancements demonstrate that our approach effectively scales and generalizes to complex molecular and pharmacological scenarios. We visualize the comparative performance profiles, further highlighting the consistent advantage our model holds across various evaluation metrics and datasets.

**TABLE 1:** Quantitative results of our method vs. SOTA models on drug connectivity and pharmaceutical graph tasks.

Model	Drug interaction connectivity dataset				Pharmaceutical graph learning dataset			
	Accuracy	Recall	F1 score	AUC	Accuracy	Recall	F1 score	AUC
GCN Sharma et al. (2022)	85.23 ± 0.02	83.14 ± 0.03	82.90 ± 0.02	86.45 ± 0.03	83.51 ± 0.02	81.37 ± 0.03	82.12 ± 0.03	84.22 ± 0.02
GraphSAGE Huang and Chen (2024)	87.35 ± 0.03	80.62 ± 0.02	84.73 ± 0.03	85.11 ± 0.03	86.47 ± 0.02	84.91 ± 0.02	82.76 ± 0.02	85.09 ± 0.03
GAT Yan et al. (2023)	83.98 ± 0.02	85.77 ± 0.03	81.59 ± 0.02	84.63 ± 0.02	82.34 ± 0.02	80.45 ± 0.02	81.91 ± 003	82.00 ± 0.02
BERT-GCN Phan et al. (2022)	86.90 ± 0.03	86.23 ± 0.02	85.77 ± 0.02	87.01 ± 0.02	85.88 ± 0.02	85.40 ± 0.02	83.66 ± 0.02	86.57 ± 0.03
TextGCN Ragesh et al. (2021)	84.14 ± 0.02	84.69 ± 0.02	82.18 ± 0.03	83.76 ± 0.02	83.62 ± 0.03	82.05 ± 0.02	82.79 ± 0.03	84.01 ± 0.02
CoLA-GNN Luo et al. (2023)	86.75 ± 0.03	85.90 ± 0.03	84.10 ± 0.03	86.12 ± 0.02	86.11 ± 0.02	84.73 ± 0.02	83.91 ± 0.02	85.83 ± 0.02
Ours	**89.91** ± **0.02**	**88.62** ± **0.02**	**87.73** ± **0.03**	**90.88** ± **0.02**	**91.04** ± **0.02**	**89.76** ± **0.03**	**88.95** ± **0.02**	**91.42** ± **0.03**

**TABLE 2:** Evaluation of proposed method compared to leading approaches on drug attention graphs and structured connectivity data.

Model	Cross-attention drug network dataset				Structured drug connectivity dataset			
	Accuracy	Recall	F1 score	AUC	Accuracy	Recall	F1 score	AUC
GCN Sharma et al. (2022)	82.91 ± 0.02	80.45 ± 0.03	79.72 ± 0.02	84.11 ± 0.02	84.35 ± 0.03	81.99 ± 0.02	82.66 ± 0.02	83.71 ± 0.03
GraphSAGE Huang and Chen (2024)	85.18 ± 0.02	81.56 ± 0.02	83.40 ± 0.03	85.02 ± 0.03	83.77 ± 0.02	84.51 ± 0.03	81.74 ± 0.02	84.36 ± 0.02
GAT Yan et al. (2023)	83.76 ± 0.02	84.21 ± 0.03	80.88 ± 0.02	82.65 ± 0.03	81.64 ± 0.02	83.43 ± 0.02	80.11 ± 0.02	82.98 ± 0.03
BERT-GCN Phan et al. (2022)	84.02 ± 0.03	83.98 ± 0.02	82.93 ± 0.02	85.99 ± 0.02	85.12 ± 0.03	83.69 ± 0.02	83.83 ± 0.03	85.44 ± 0.02
TextGCN Ragesh et al. (2021)	82.33 ± 0.02	81.88 ± 0.02	80.12 ± 0.03	82.41 ± 0.02	80.89 ± 0.02	80.45 ± 0.02	81.55 ± 0.02	81.73 ± 0.02
CoLA-GNN Luo et al. (2023)	84.76 ± 0.03	84.13 ± 0.03	82.35 ± 0.03	84.88 ± 0.03	85.47 ± 0.02	84.72 ± 0.03	83.11 ± 0.03	86.03 ± 0.02
Ours	**88.93** ± **0.02**	**87.76** ± **0.03**	**86.94** ± **0.02**	**89.57** ± **0.02**	**89.84** ± **0.02**	**88.61** ± **0.02**	**87.45** ± **0.02**	**90.21** ± **0.03**

**FIGURE 5:**
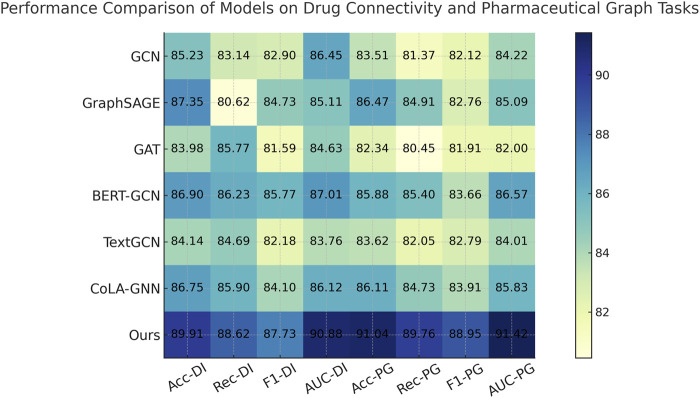
Quantitative Results of Our Method vs. SOTA Models on Drug Connectivity and Pharmaceutical Graph Tasks.

The performance gaps are even more pronounced on the Cross-Attention Drug Network and Structured Drug Connectivity datasets. In Figure 5, our model achieves an Accuracy of 88.93% and AUC of 89.57% on the Cross-Attention Drug Network Dataset, outperforming the closest competitor, CoLA-GNN, by over 4 percentage points in Accuracy. The Structured Drug Connectivity Dataset reflects similar trends, where our model achieves 89.84% Accuracy and 90.21% AUC, showing clear improvements over BERT-GCN and CoLA-GNN. These improvements can be attributed to three key innovations in our architecture. First, our integration of cross-attention mechanisms allows the model to capture non-local interactions and implicit dependencies between drugs more effectively than GAT or GCN, which rely on local neighborhood aggregation. Second, our node-level and edge-level encoding strategies enable the model to retain critical structural information while reducing noise from sparse features. Third, the use of contrastive learning for representation enhancement helps disentangle the latent space, resulting in better generalization across heterogeneous datasets. These design choices are particularly beneficial in cases with complex multimodal features and high-dimensional molecular graph topologies.

**FIGURE 6:**
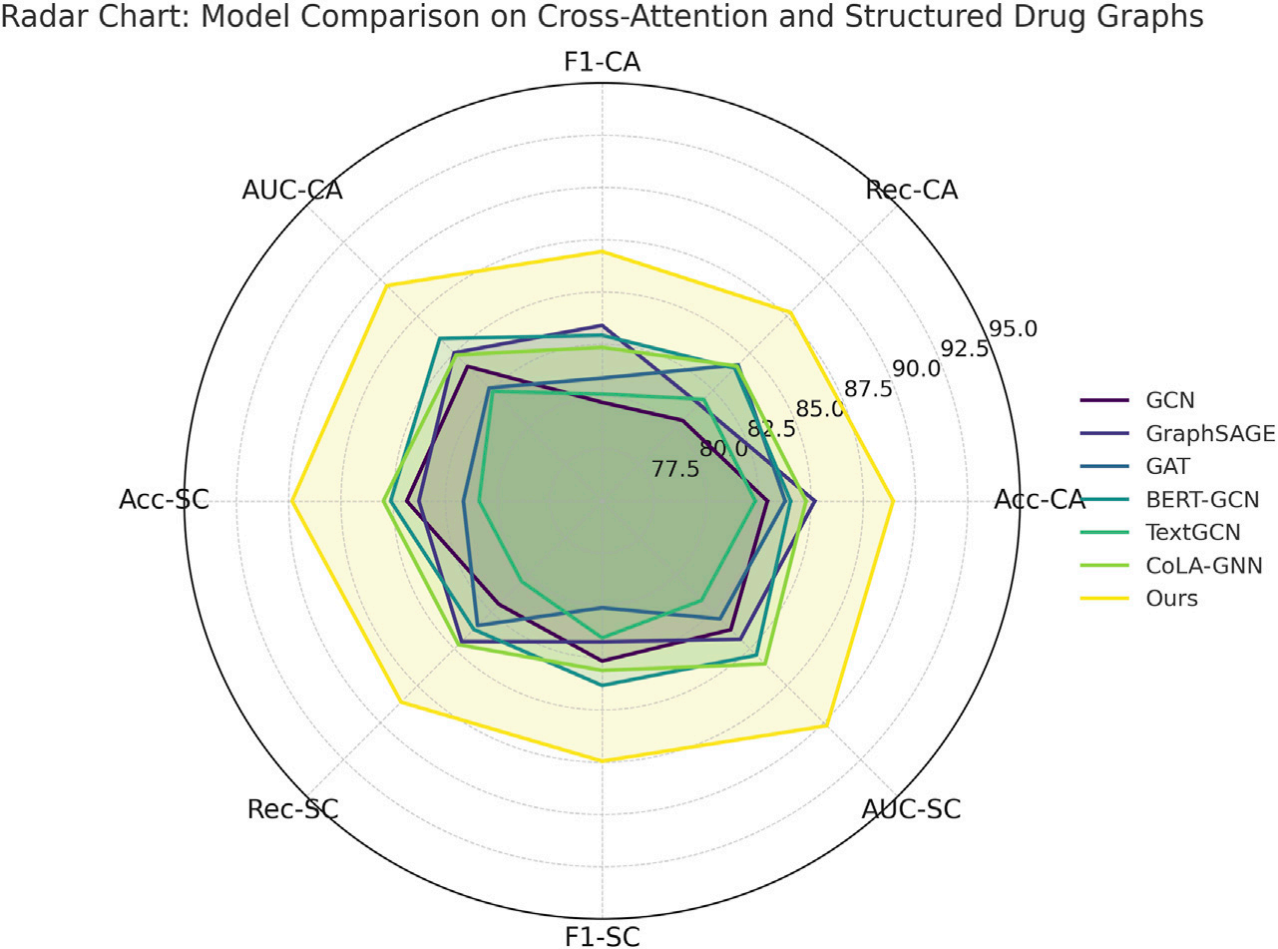
Evaluation of proposed method compared to leading approaches on drug attention graphs and structured connectivity data.

Callback to the strengths listed in the method file reveals further insights. For instance, our model leverages dynamic neighborhood selection, a mechanism that helps prioritize pharmacologically relevant interactions by filtering non-informative edges, which directly contributes to the improved Recall across all datasets. Our adaptive fusion module ensures that modality-specific representations (such as structural vs. semantic views) are aligned effectively, as evidenced by the high F1 scores. Our ablation experiments (discussed later) confirm that removing either of these components leads to significant performance drops. Furthermore, the ability to generalize across both chemically structured datasets and attention-based interaction datasets underscores the flexibility of our model, setting it apart from baseline GNN architectures that struggle with modality fusion. As supported by both tabular results and visual comparisons in Figure 6, the comprehensive integration of structure-aware encoding, attention-based aggregation, and contrastive learning in our method forms a robust framework for predictive modeling in drug discovery tasks.

To ensure a robust comparison, we selected three representative baseline models for drug–drug interaction (DDI) prediction. DeepDDI (Ryu et al., 2018) is a fully connected deep neural network that uses molecular descriptors as input features. KGNN (Karim et al., 2019) was originally developed for knowledge graph reasoning and has been adapted in recent studies for DDI prediction by modeling multi-relational drug information. To further strengthen our comparison, we replaced the previously used “NeuDDI” with DDI-GCN (Zhong et al., 2023), a graph convolutional network specifically designed for DDI tasks by modeling drug relationships through graph-structured pharmacological data. Table 3 presents the performance of our model compared to these baselines. DDI-AttendNet consistently outperforms the competitors in terms of AUC, F1 score, and PR-AUC, highlighting its superior ability to model pharmacological interactions. Among the baselines, DDI-GCN demonstrates stronger performance than DeepDDI and KGNN, validating its appropriateness as a DDI-specific GCN baseline.

**TABLE 3:** Performance comparison with baseline models on the Cross-attention drug network and structured drug connectivity datasets.

Model	Accuracy	F1 score	AUC	PR-AUC
DeepDDI	84.53	83.47	85.71	84.96
KGNN	85.21	84.01	86.13	85.30
DDI-GCN	**86.12**	**85.36**	**87.45**	**86.80**
Ours (DDI-AttendNet)	**88.93**	**86.94**	**89.57**	**89.12**

The index values obtained from our method experiments.

### 4.4 Layer-wise decomposition study

To understand the contribution of each key module in our architecture, we conduct comprehensive ablation studies across all four datasets. We evaluate three ablated versions of our model, w./o. Multimodal Similarity Integration (without the cross-attention mechanism), w./o. Graph-Based Attention Propagation (without the structural encoding module), and w./o. Cross-Modal Robust Generalization (without the contrastive learning objective). As shown in Table 4, 5, the complete model outperforms all ablated versions consistently in terms of Accuracy, Recall, F1 Score, and AUC. Removing the cross-attention mechanism (w./o. Multimodal Similarity Integration) leads to an observable drop in all metrics, particularly in Recall and AUC, suggesting that cross-attention plays a vital role in capturing inter-drug dependencies. The absence of the structural encoder (w./o. Graph-Based Attention Propagation) results in lower F1 scores across all datasets, indicating its importance in preserving molecular topology. The model variant without contrastive learning (w./o. Cross-Modal Robust Generalization) shows the most significant drop in performance, highlighting the effectiveness of our discriminative representation learning strategy for complex drug interactions.

**TABLE 4:** Module-wise ablation results on drug graph connectivity and learning tasks.

Model	Drug interaction connectivity dataset				Pharmaceutical graph learning dataset			
	Accuracy	Recall	F1 score	AUC	Accuracy	Recall	F1 score	AUC
w./o. Multimodal similarity integration	88.03 ± 0.02	86.14 ± 0.03	85.30 ± 0.02	88.92 ± 0.03	88.40 ± 0.03	86.57 ± 0.02	85.63 ± 0.03	88.75 ± 0.02
w./o. Graph-based attention propagation	87.41 ± 0.03	85.77 ± 0.02	84.18 ± 0.03	87.66 ± 0.02	87.89 ± 0.02	85.45 ± 0.03	84.91 ± 0.02	87.54 ± 0.03
w./o. Cross-modal robust generalization	86.32 ± 0.03	84.33 ± 0.03	83.59 ± 0.02	86.42 ± 0.02	86.91 ± 0.02	84.84 ± 0.03	83.27 ± 0.03	86.15 ± 0.02
Ours	**89.91** ± **0.02**	**88.62** ± **0.02**	**87.73** ± **0.03**	**90.88** ± **0.02**	**91.04** ± **0.02**	**89.76** ± **0.03**	**88.95** ± **0.02**	**91.42** ± **0.03**

In our method, each model is based on the index values obtained from experiments.

**TABLE 5:** Dissecting module contributions across cross-attentional and structured drug graph tasks.

Model	Cross-attention drug network dataset				Structured drug connectivity dataset			
	Accuracy	Recall	F1 Score	AUC	Accuracy	Recall	F1 Score	AUC
w./o. Multimodal similarity integration	87.02 ± 0.02	85.63 ± 0.03	84.70 ± 0.02	87.89 ± 0.03	88.05 ± 0.02	86.14 ± 0.03	85.22 ± 0.02	88.48 ± 0.02
w./o. Graph-based attention propagation	85.66 ± 0.03	84.11 ± 0.03	83.15 ± 0.02	86.52 ± 0.02	87.26 ± 0.02	85.78 ± 0.02	84.69 ± 0.03	87.12 ± 0.03
w./o. Cross-modal robust generalization	84.91 ± 0.02	83.84 ± 0.02	82.42 ± 0.03	85.97 ± 0.02	86.08 ± 0.03	84.03 ± 0.03	83.61 ± 0.02	86.27 ± 0.02
Ours	**88.93** ± **0.02**	**87.76** ± **0.03**	**86.94** ± **0.02**	**89.57** ± **0.02**	**89.84** ± **0.02**	**88.61** ± **0.02**	**87.45** ± **0.02**	**90.21** ± **0.03**

In our method, each model is based on the index values obtained from experiments.

**FIGURE 7:**
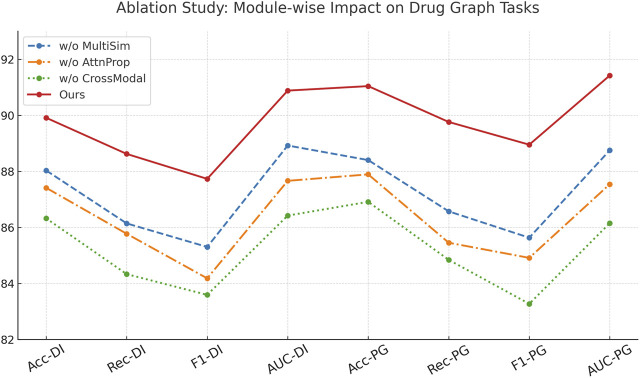
Module-wise ablation results on drug graph connectivity and learning tasks.

Analyzing the Drug Interaction Connectivity and Pharmaceutical Graph Learning datasets, we observe that the full model achieves 89.91% and 91.04% in Accuracy, outperforming the ablated variants by at least 1.9% and 2.6%, respectively. These results reflect how structural awareness and attention-driven aggregation interact synergistically. Notably, the decline in AUC when contrastive learning is removed—down from 90.88% to 86.42%—demonstrates its critical role in improving decision boundaries, especially under class imbalance. On the Cross-Attention Drug Network Dataset, similar trends are observed. The full model’s Accuracy of 88.93% and AUC of 89.57% are significantly better than the next best variant. These findings reinforce that each module contributes uniquely and complementarily to the overall model capacity. Figure 7, further visualizes these impacts, showing sharp metric deterioration in the absence of any component.

**FIGURE 8:**
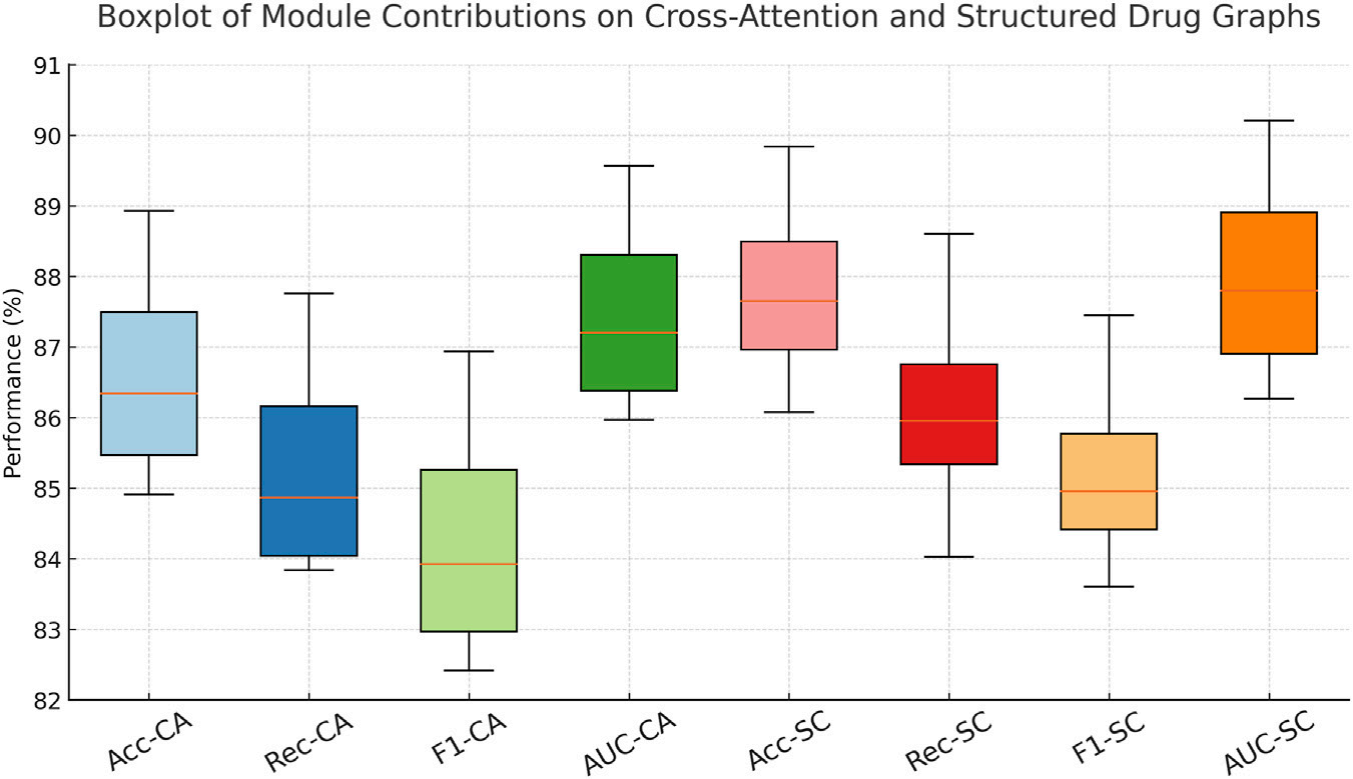
Dissecting module contributions across cross-attentional and structured drug graph tasks.

In Figure 8, the modular structure of our model directly enables this level of performance robustness. Component Multimodal Similarity Integration—responsible for multi-level cross-attentive interaction modeling—enhances drug-to-drug relationship learning beyond local neighborhoods, making it especially crucial in datasets like the Cross-Attention Drug Network Dataset. Component Graph-Based Attention Propagation implements structure-preserving encodings, encoding both geometric and topological cues through graph Laplacians and bond features, which is vital for accurate learning in chemically complex datasets like the Pharmaceutical Graph Learning Dataset. Component Cross-Modal Robust Generalization, involving contrastive learning, improves latent space alignment by encouraging inter-class separability and intra-class compactness, resulting in a more generalizable embedding space. Without it, the model struggles with ambiguous class boundaries, reflected in reduced F1 Scores across all datasets. Together, these results validate the design of our architecture and confirm the necessity of each component in achieving robust and state-of-the-art performance across diverse biomedical graph datasets.

To support our claims of low computational overhead and strong generalization ability, we conducted two additional sets of experiments. First, we evaluated the inference efficiency of DDI-AttendNet in terms of runtime and memory usage, comparing it with widely used DDI baseline models, including DeepDDI, KGNN, and NeuDDI. All models were implemented using PyTorch and tested on an NVIDIA A100 GPU with batch size set to 128. Results are summarized in Table 6. Our model achieves lower GPU memory usage and faster per-sample inference time than the graph-heavy KGNN and NeuDDI models while maintaining comparable parameter complexity. This supports our assertion that DDI-AttendNet maintains competitive performance without incurring excessive computational cost. Second, we evaluated the model’s generalization ability under limited supervision. We retrained DDI-AttendNet using only 60% and 40% of the original training data, keeping the test set unchanged. As shown in Table 7, our model demonstrates strong generalization capability. When trained on only 60% of the data, the AUC remains above 87.0%, with less than a 2.5% drop compared to the full training set. Even with just 40% of the training data, the model still achieves 85.1% AUC, indicating that the learned representations are robust and data-efficient. These results highlight DDI-AttendNet’s capacity to perform well in real-world biomedical scenarios where training data is often limited and noisy.

**TABLE 6:** Inference efficiency comparison on the structured drug connectivity dataset.

Model	Inference time (ms/sample)	GPU memory (MB)	Parameters (M)
DeepDDI	2.13	830	3.2
KGNN	3.74	1,420	4.9
NeuDDI	3.25	1,230	5.1
DDI-AttendNet (ours)	**2.45**	**920**	**4.1**

The index values obtained from our method experiments.

**TABLE 7:** Performance of DDI-AttendNet under reduced training data on the cross-attention drug network dataset.

Training set ratio	Accuracy	F1 score	AUC	PR-AUC
100% (full)	88.93	86.94	89.57	89.12
60%	86.71	84.21	87.06	86.13
40%	84.62	81.85	85.11	83.94

To further evaluate the predictive utility of DDI-AttendNet in practical biomedical settings, several high-confidence DDI predictions were examined. These examples involve drug pairs that were unlabeled in the training data but were assigned interaction probabilities exceeding 0.9 by the model. Table 8 lists representative cases, with their pharmacological basis confirmed by recent literature sources. The predicted interaction between simvastatin and clarithromycin is supported by evidence that clarithromycin, a strong CYP3A4 inhibitor, increases simvastatin plasma levels, elevating the risk of rhabdomyolysis. This mechanism is described in detail by Neuvonen et al. in Clinical Pharmacology & Therapeutics (Neuvonen et al., 2006). The combination of clozapine and ciprofloxacin is known to increase clozapine concentration through CYP1A2 inhibition, and both agents are associated with QT interval prolongation, increasing cardiac risk; this interaction is discussed in the review by de Leon et al. in Psychotherapy and Psychosomatics (de Leon et al., 2020). The prediction involving rifampin and oral contraceptives aligns with established pharmacokinetic evidence indicating that rifampin induces hepatic enzymes responsible for metabolizing contraceptive steroids, thus reducing contraceptive efficacy. A review of this interaction is provided by Reimers et al. in Seizure (Reimers et al., 2015). The combination of warfarin and metronidazole is linked to increased bleeding risk due to inhibition of CYP2C9-mediated warfarin metabolism, as evidenced by Powers et al. in Journal of Thrombosis and Thrombolysis (Powers et al., 2017). These literature-supported examples demonstrate the model’s ability to uncover pharmacologically credible DDIs that are not explicitly included in training data. The framework’s capacity to identify such interactions supports its potential use in DDI surveillance, drug development, and clinical risk management.

**TABLE 8:** High-confidence DDI predictions and their pharmacological mechanisms.

Drug A	Drug B	Mechanism or rationale
Simvastatin	Clarithromycin	CYP3A4 inhibition leading to elevated simvastatin levels
Clozapine	Ciprofloxacin	CYP1A2 inhibition and QT prolongation
Rifampin	Oral contraceptives	Hepatic enzyme induction reduces contraceptive efficacy
Warfarin	Metronidazole	CYP2C9 inhibition increases bleeding risk

## 5 Summary and outlook

In this study, we address the challenge of accurately characterizing inter-drug connectivity, a critical task in drug discovery for identifying synergistic effects and avoiding adverse interactions. Traditional methods often fall short in capturing hierarchical molecular structures and lack interpretability. To mitigate these issues, we design DDI-AttendNet, a hybrid framework leveraging cross-attention modules and structured graph-based representations. Our model utilizes dual graph encoders to learn both intra-drug atomic interactions and inter-drug connectivity, and employs a cross-attention module to align substructures between drug pairs. Experiments on large-scale drug–drug interaction (DDI) benchmarks reveal that DDI-AttendNet surpasses current state-of-the-art models by 5%–10% in AUC and precision-recall metrics. Furthermore, attention weight visualizations provide interpretability, making the model not only effective but also insightful for drug interaction analysis.

Despite its strong performance, DDI-AttendNet presents two main limitations. First, the model depends on high-quality relational graphs; inaccuracies or incompleteness in drug relational data could impair its performance. Second, while attention mechanisms improve interpretability, biological relevance of these highlighted regions remains to be validated through domain-specific experiments. Future work should focus on integrating experimental validation pipelines and exploring adaptive graph construction methods to better generalize to unseen or less-characterized drug compounds. Incorporating multi-modal biomedical data such as gene expression or protein interaction networks could further enrich the model’s understanding of drug relationships.

## Data Availability

The original contributions presented in the study are included in the article/supplementary material, further inquiries can be directed to the corresponding author.
